# Revisão sobre fraturas osteoporóticas: Ocorrência, prevenção e consequências

**DOI:** 10.1055/s-0045-1809515

**Published:** 2025-06-14

**Authors:** Nelson Elias, José Eduardo Grandi Ribeiro, Luiz Augusto Campinho, Cilas Reis, Luiz Arthur Miguelote S. Elias, Pedro José Labronici

**Affiliations:** 1 Universidade Estadual Rio de Janeiro, Rio de Janeiro, RJ, Brasil; 2 Universidade Federal Rio de Janeiro, Rio de Janeiro, RJ, Brasil; 3 Escola de Ciências Médicas, Santa Casa de Misericórdia de Vitória (EMESCAM), Vitória, ES, Brasil; 4 Serviço de Ortopedia e Traumatologia, Hospital Estadual de Urgência e Emergência de Vitória (HEUE), Vitória, ES, Brasil; 5 Faculdade de Medicina, Universidade Vila Velha, Vila Velha, ES, Brasil; 6 Universidade Federal Fluminense (UFF), Niterói, RJ, Brasil

**Keywords:** avaliação de risco, diagnóstico, fraturas por estresse, osteoporose, tratamento, diagnosis, fractures, stress, osteoporosis, risk assessment, treatment

## Abstract

A osteoporose é uma condição metabólica que compromete a densidade e a arquitetura ósseas, o que aumenta o risco de fraturas e tem impacto na morbimortalidade. O diagnóstico envolve a densitometria óssea, em que se avalia a densidade mineral em áreas propensas a fraturas. A osteoporose primária, relacionada à idade, pode permanecer assintomática por anos, ao passo que a secundária resulta de comorbidades ou medicamentos. Cerca de 80% das mulheres brancas na pós-menopausa têm osteoporose, com expectativa de aumento com o envelhecimento. Tratamentos ortopédicos são comuns para fraturas, que são frequentemente causadas por quedas em idosos. A prevenção de fraturas é crucial, e requer políticas de saúde pública e terapias com este objetivo.

## Abstract (en)

Osteoporosis is a metabolic condition that compromises bone density and architecture, increasing the risk of fractures and impacting morbidity and mortality. The diagnosis involves bone densitometry, in which mineral density in areas prone to fractures is assessed. Primary osteoporosis is age-related and may remain asymptomatic for years, while secondary osteoporosis results from comorbidities or medications. Approximately 80% of postmenopausal Caucasian women have osteoporosis, with an expected increase with aging. Orthopedic treatments are common for fractures, which are often caused by falls in the elderly. Fracture prevention requires public health policies and therapies focused on this goal.

## Introdução

### Definição

A osteoporose é caracterizada pela diminuição da densidade e da qualidade ósseas, o que aumenta o risco de fraturas devido à porosidade e à fragilidade dos ossos. A perda óssea é gradual e assintomática, e muitas vezes é denominada “doença silenciosa”. As fraturas, que ocorrem frequentemente na coluna, no punho, no quadril e no ombro, costumam ser o primeiro sintoma evidente da osteoporose. 1 2

### Epidemiologia

A osteoporose é um sério problema de saúde pública global, pois afeta cerca de 200 milhões de mulheres em todo o mundo, com incidência crescente nas faixas etárias mais avançadas. 1 2 3 4 Aproximadamente 30% das mulheres na pós-menopausa nos Estados Unidos e na Europa têm osteoporose, e uma significativa proporção sofrerá fraturas por fragilidade ao longo da vida. 2 O envelhecimento da população contribuirá para um aumento substancial na incidência de osteoporose, com projeções que indicam aumentos expressivos nas taxas de fraturas de quadril até 2050 em comparação com 1990. 3

### Custos de Saúde

A osteoporose impacta mais de 10 milhões de adultos nos Estados Unidos, acarreta elevados custos sociais (22 bilhões de dólares em 2008), e é subdiagnosticada e subtratada. Projeções indicam um aumento no número anual de fraturas de 1,9 milhões para 3,2 milhões até 2040, com custos associados que ultrapassam os 95 bilhões de dólares. Intervenções relacionadas à osteoporose podem reduzir fraturas e custos. 4 5 6 7 8 O ônus econômico supera o de condições como enxaqueca, e se assemelha ao da artrite reumatoide. O uso contínuo de medicamentos para osteoporose está associado à redução de riscos de fraturas e custos de saúde. Prevê-se que os custos médicos diretos alcancem 25,3 bilhões de dólares até 2025, e isso põe em destaque a importância da melhoria na persistência da medicação para pagadores e pacientes. 4 5

### Fatores de Risco

Os fatores de risco para o desenvolvimento de osteoporose podem ser classificados como permanentes (como idade e gênero) e modificáveis (como tabagismo, consumo de álcool e hábitos alimentares). Esses fatores aumentam a probabilidade de desenvolver a doença, e a presença de múltiplos fatores aumenta o risco. Apresentar fatores de risco não leva necessariamente ao desenvolvimento da osteoporose, mas quanto mais fatores e quanto mais intensos eles forem, maior será a probabilidade de ocorrência. 2 5 6 7 8 9

### História da Pesquisa em Osteoporose

A osteoporose, uma condição que afeta os ossos, foi identificada oficialmente há cerca de três séculos, embora tenha existido por milênios. A descoberta do processo de remodelação óssea por John Hunter há 250 anos foi crucial para entender como o tecido ósseo é removido e substituído. 10 O cirurgião Astley Cooper relacionou o declínio da densidade óssea ao aumento do risco de fraturas. O termo *osteoporose* foi introduzido por Jean Lobstein na década de 1830. 11 12

No início do século XX, Kyes e Potter 13 observaram a relação entre níveis de estrogênio e densidade óssea em pombos. Mais tarde, Fuller Albright et al. 14 15 contribuíram significativamente para a compreensão da osteoporose, ao identificar uma deficiência de osteoblastos e associá-la à menopausa em mulheres. Eles desenvolveram o primeiro tratamento eficaz com o uso de estrogênio.

Em 1955, Alexander Cooke 16 definiu a osteoporose como uma formação óssea inadequada devido à falta de “matriz”, e sugeriu que o diagnóstico deveria basear-se em exames histológicos. Cooke 16 também mencionou os efeitos benéficos e adversos dos andrógenos. Ensaios terapêuticos subsequentes tiveram sucesso limitado, ao utilizar flúor, esteroides anabolizantes e calcitonina, mas apresentaram alguns efeitos colaterais. 15 16 17 18

## Patogênese

A osteoporose pode ocorrer devido à falha em atingir o pico de massa óssea e à reabsorção óssea excessiva e/ou diminuição da formação óssea durante os processos de remodelação. Todos esses processos provavelmente contribuem para a osteoporose, em diferentes níveis. 19

### Pico de Massa Óssea

O pico de massa óssea é o estágio da vida em que a densidade mineral óssea (DMO) atinge seu ponto máximo. A massa óssea aumenta durante a infância e a adolescência, e atinge esse pico por volta dos 30 anos. Após atingir o pico de massa óssea, a DMO tende a diminuir gradualmente com o envelhecimento. É crucial para prevenir a osteoporose e as fraturas na vida adulta. Um aumento de 10% no pico de massa óssea pode resultar em uma redução de 30% nas fraturas de quadril. Fatores genéticos desempenham um papel significativo, pois contribuem com cerca de 80% da variabilidade do pico de massa óssea, conforme evidenciado por estudos com gêmeos. Variantes genéticas, como a proteína-5 relacionada a receptor de lipoproteína de baixa densidade ( *low-density lipoprotein receptor-related protein 5* , LRP5, em inglês), 5 a esclerostina e outras, foram identificadas por estudos de associação de todo o genoma. 19 Além disso, fatores ambientais, como nutrição, exercícios e tabagismo, desempenham papéis importantes no desenvolvimento do pico de massa óssea. 19 A modulação desse pico pode ocorrer durante a vida intrauterina, e é afetada pela nutrição materna, pelo tabagismo e pelos níveis de exercício. 20

### Reabsorção Óssea e Desequilíbrio de Formação

O processo de remodelação óssea, que envolve a ação coordenada de osteoclastos e osteoblastos, é crucial para a manutenção da saúde óssea na vida adulta, pois repara microdanos. Embora o aumento da reabsorção óssea pareça influenciar a perda óssea e o risco de fraturas, a formação óssea comprometida também desempenha um papel importante na osteoporose. 21 Este comprometimento resulta em parte de um número reduzido de células osteoprogenitoras/pré-osteoblásticas e/ou de um defeito relacionado à idade na transformação de células estromais em adipócitos em vez de osteoblastos. As perdas ósseas associadas à idade e à menopausa são determinantes significativos para a osteoporose, e são fatores genéticos responsáveis por variações na integridade esquelética entre idosos da mesma faixa etária. 22 23

### Doenças Associadas à Osteoporose

Indivíduos com determinados problemas de saúde, que resultam em maior perda óssea e/ou risco de quedas, apresentam um risco maior de desenvolver osteoporose. 24 25 A osteoporose secundária é causada por diversas comorbidades e/ou medicamentos ( Tabela 1 ), e está associada a doenças que prejudicam os mecanismos relacionados ao equilíbrio de cálcio, vitamina D e hormônios sexuais. 26 Cerca de um terço das mulheres na pós-menopausa, assim como muitos homens e mulheres na pré-menopausa, apresentam causas concomitantes de perda óssea, 25 como a hipercalciúria renal, que é tratável com diuréticos tiazídicos. 26 O tratamento é específico para cada doença associada, e requer abordagem multidisciplinar. 25

**Tabela 1:** Causas da osteoporose secundária em adultos 25

Artrite reumatoide e outras condições reumatológicas
Síndromes de má absorção
Deficiência de hormônio sexual
Hiperparatireoidismo primário
Doença renal crônica
Doença hepática crônica
Diabetes
Doença pulmonar obstrutiva crônica
Hipertireoidismo não tratado
Problemas neurológicos
Câncer

### Metabolismo e Regulação Óssea em Pacientes com Osteoporose

Os ossos passam por remodelações constantes, em um equilíbrio entre a formação pelos osteoblastos e a reabsorção pelos osteoclastos. 9 A osteoporose, caracterizada pela redução da densidade óssea, aumenta o risco de fraturas. 16 17 21 A doença óssea metabólica abrange anomalias causadas por distúrbios genéticos ou deficiências de minerais como cálcio, fósforo e vitamina D, e inclui condições como osteoporose, osteomalácia, raquitismo e doença óssea de Paget. 27

A ingestão inadequada de cálcio e a deficiência de vitamina D estão associadas à osteoporose, 28 e níveis adequados de vitamina D ajudam a manter a resistência óssea. Apesar disso, a terapia com cálcio e vitamina D pode não prevenir completamente a perda óssea, e a terapia hormonal à base de estrogênio e progesterona, embora retarde a osteoporose, apresenta efeitos colaterais. Testes de metabolismo ósseo podem oferecer alternativas terapêuticas.

A orientação das doses de suplementação de cálcio e vitamina D é determinada pelos níveis sanguíneos. Indicadores como cálcio na urina e testes hormonais auxiliam na avaliação da absorção intestinal e na determinação da origem da osteoporose (primária ou secundária). 28

## Cálcio e Fósforo no Metabolismo Corporal

Os níveis de cálcio ósseo e sanguíneo, essenciais para o equilíbrio fisiológico, são dinâmicos e regulados por hormônios como a vitamina D, a calcitonina e o hormônio da paratireoide ( *parathyroid hormone* , PTH, em inglês). A vitamina D facilita a absorção intestinal de cálcio, ao manter níveis adequados para a mineralização óssea e a prevenção de tetania hipocalcêmica. A deficiência de vitamina D pode resultar em ossos frágeis e deformados, ao passo que a suficiência previne raquitismo em crianças e osteomalácia em adultos, e ajuda a prevenir a osteoporose em idosos. 28 29 30 31

A osteoporose, que está relacionada à deterioração da microarquitetura óssea, pode ser avaliada por marcadores de renovação óssea ( *bone turnover markers* , BTMs, em inglês), que complementam a medição da DMO na avaliação dos riscos de fratura. Os hormônios sexuais, especialmente o estrogênio, desempenham um papel crucial na osteoporose primária, e a deficiência de estrogênio na menopausa é uma causa importante. A falta de estrogênio afeta diretamente os osteoclastos, o que ocasiona distúrbios ósseos, incluindo a osteoporose. 32 33

### Avaliação da Densidade Mineral Óssea

**Figs. 1 e 2:**
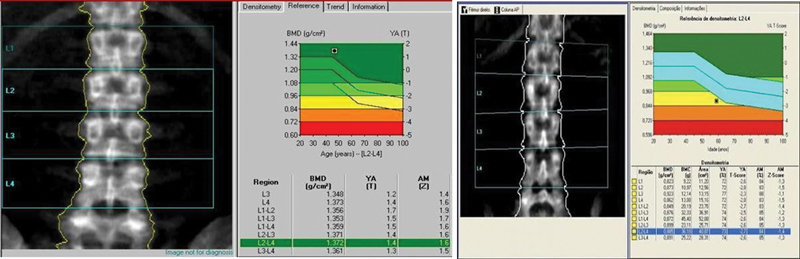
Densidade mineral óssea (DMO) de idosa saudável ( Fig. 1 ) e de idosa com osteoporose ( Fig. 2 ). A Fig. 1 mostra um teste de DMO de uma mulher idosa saudável. A radiografia mostra ossos do quadril normais. O gráfico mostra o valor da DMO na zona verde (normal). A Fig. 2 mostra um teste de DMO de uma mulher idosa com osteoporose. A radiografia mostra ossos do quadril mais fracos. O gráfico mostra o valor da DMO na zona vermelha (osteoporose), o que coloca a paciente em risco muito maior de sofrer fraturas.

A pontuação Z na densitometria óssea indica o número de desvios padrão acima ou abaixo do esperado para alguém da mesma idade, sexo, peso e origem étnica ou racial que o paciente avaliado. Uma pontuação Z baixa sugere perda óssea anormal, ao passo que uma pontuação alta é normal. Esta pontuação não é atribuível ao envelhecimento, e pode indicar um problema subjacente tratável para retardar ou interromper a perda óssea 34 35 36 37 ( Figs. 1,2 ).

O raio X é um exame médico não invasivo amplamente utilizado para diagnosticar e tratar condições médicas, e é o método de imagem mais antigo. Pode examinar o corpo inteiro ou partes específicas, e utiliza doses baixas de radiação ionizante. Em alguns casos, dispositivos periféricos de raios X ou ultrassom são usados para avaliar massa óssea baixa, e a tomografia computadorizada (TC) com *software* especial também pode diagnosticar ou monitorar massa óssea baixa, embora seja menos utilizada do que a varredura de LRP por absorciometria de raios X de dupla energia ( *dual-energy X-ray absorptiometry* , DXA, em inglês). 36 37

### Tipos de Fratura

A osteoporose é uma condição assintomática, frequentemente diagnosticada após a ocorrência de fraturas, que são a principal manifestação da doença e evidenciam atrofia óssea. Quando relacionada à idade, a osteoporose é subdiagnosticada por permanecer silenciosa durante vários anos até a ocorrência de fraturas, especialmente em idosos, o que resulta em limitações nas atividades diárias. Fraturas osteoporóticas podem levar a complicações graves e aumentar as taxas de morbidade e mortalidade. 9 10 Pacientes adultos com fraturas devem ser avaliados quanto à osteoporose, uma vez que cerca de 30% dos casos apresentam causas secundárias, notadamente nos casos de mulheres na pré-menopausa, homens com osteoporose e pacientes com fratura de quadril. Testes laboratoriais apropriados são úteis para investigar causas secundárias. 3 9 10 11

A incidência crescente de fraturas em idosos tornou-se um problema de saúde significativo em vários países, com a expectativa de aumento devido ao envelhecimento da população. Mais de 90% das fraturas são causadas por quedas de baixa energia, o que resulta em elevadas taxas de mortalidade. Na Austrália, por exemplo, prevê-se que a incidência de fraturas de quadril aumentará de 20 mil para 50 mil casos em 2050 devido ao envelhecimento da população. 8 9 11 12

**Figs. 3 A,B e 4 A,B:**
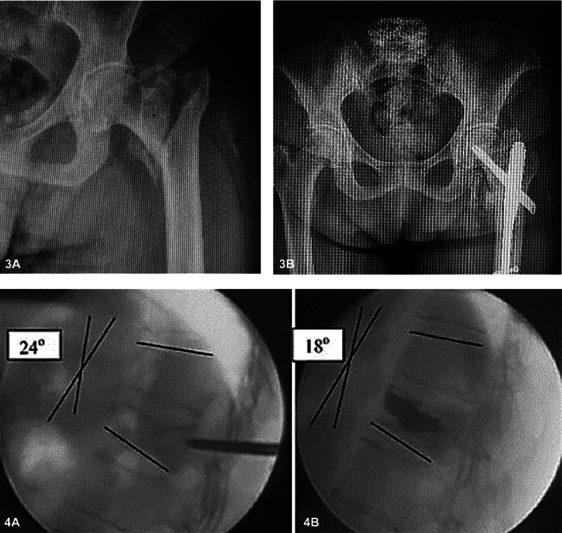
Exemplos dos dois locais mais comuns acometidos por fraturas osteoporóticas e seus tratamentos ortopédicos. ( **3A** ) Pré-operatório de fratura transtrocantérica. ( **3B** ) Tratamento cirúrgico. ( **4A** ) Pré-operatório de fratura vertebral. ( **4B** ) Tratamento cirúrgico após cifoplastia (injeção intraóssea de cimento ortopédico). A cifose angular foi reduzida de 24 para 18 graus.

As fraturas osteoporóticas, frequentemente resultantes de quedas, afetam principalmente vértebras, quadris, punhos e ombros. ( Figs. 3,4 ) A osteoporose afeta cerca de 80% das mulheres brancas na pós-menopausa, e terá um impacto crescente com o envelhecimento da população. 11 12 13 Essas fraturas, especialmente as de quadril e de vértebra, têm uma mortalidade de até 20% em 12 meses, devido à hospitalização e ao aumento do risco de complicações. O diagnóstico muitas vezes ocorre apenas após a primeira fratura, que geralmente requer tratamento cirúrgico ortopédico. 4 5 11

A Ferramenta de Avaliação de Risco de Fratura (Fracture Risk Assessment Tool, FRAX, em inglês) é validada, e calcula o risco de fratura em 10 anos com base em fatores individuais, incluindo a DMO. Amplamente integrada a diretrizes nacionais, a FRAX é acessível, fácil de usar para profissionais de saúde, e útil, considerando o aumento exponencial nas taxas de fraturas de quadril com a idade. 4 11 12 38

### Identificação e Determinação do Risco de Fratura

A osteoporose aumenta o risco de fraturas devido à baixa DMO, microarquitetura/mineralização óssea prejudicada e/ou resistência óssea reduzida. Geralmente assintomática, é frequentemente diagnosticada após fraturas de baixo trauma no quadril, na coluna vertebral, no úmero proximal, na pelve ou no punho, que podem requerer hospitalização, e representam um fardo econômico significativo para os sistemas de saúde. Os fatores de risco incluem características permanentes, como idade e sexo, e modificáveis, relacionadas ao estilo de vida pessoal. 4 5 6 7 8 9

### Riscos Permanentes

Os fatores de risco permanentes, como idade, gênero feminino, histórico familiar de osteoporose, fratura anterior, etnia, menopausa/histerectomia, terapia com glicocorticoides de longo prazo, artrite reumatoide e hipogonadismo primário/secundário em homens, não podem ser modificados. No entanto, é importante que os indivíduos estejam cientes desses fatores para tomar medidas que possam ajudar a reduzir a perda mineral óssea. Além disso, existem “fatores de risco secundários” relacionados a distúrbios e medicamentos que enfraquecem os ossos, que aumentam o risco de fraturas devido a quedas. 2 5 6

### Riscos Modificáveis

Os fatores de risco modificáveis, como álcool, tabagismo, baixo índice de massa corporal, nutrição deficiente, deficiência de vitamina D, distúrbios alimentares, falta de exercício, baixa ingestão de cálcio na dieta e quedas frequentes, afetam diretamente a DMO e aumentam o risco de fraturas. 7 8 O diagnóstico de osteoporose é geralmente realizado por meio de medidas de DMO, especialmente no quadril e na coluna lombar, utilizando o aparelho de DXA. A FRAX, que considera fatores como idade, raça, consumo de álcool, sexo, índice de massa corporal, histórico de tabagismo, histórico de fraturas e medições de DMO, é uma ferramenta eficaz para prever o risco de fraturas osteoporóticas em 10 anos.

## Terapia

a) Evidência de terapia medicamentosa médica.
b) Manejo não Farmacológico e atividade física evidência de terapia medicamentosa médica.

O tratamento varia de acordo com a causa da osteoporose, e o tratamento da osteoporose secundária é mais complexo e está diretamente relacionado à doença subjacente. A terapia farmacológica visa reduzir o risco de fraturas, e muitos métodos utilizados no tratamento também são empregados na prevenção. 1 2

A osteoporose primária, muitas vezes ligada à idade e à deficiência de hormônios sexuais, resulta na deterioração contínua das trabéculas ósseas. Na osteoporose relacionada à idade, a produção reduzida de estrogênio em mulheres na pós-menopausa leva a uma significativa perda óssea. A globulina de ligação de hormônios sexuais, que inativa testosterona e estrogênio em homens durante o envelhecimento, pode contribuir para a diminuição da DMO ao longo do tempo. 37 38 39

A osteoporose secundária é ocasionada por várias comorbidades e/ou medicamentos. As doenças relacionadas frequentemente afetam os mecanismos ligados ao desequilíbrio de cálcio, vitamina D e hormônios sexuais. Por exemplo, a síndrome de Cushing, caracterizada pelo excesso de produção de glicocorticoides, pode acelerar a perda óssea. Os glicocorticoides são os medicamentos mais comuns associados à osteoporose induzida por medicamentos, com uma redução rápida nos níveis ósseos dentro de 3 a 6 meses após o início da terapia. 39 40

A osteoporose secundária apresenta causas distintas entre os sexos. Em homens, o consumo excessivo de álcool, o uso de glicocorticoides e o hipogonadismo são mais comuns. Em mulheres, 32,4% dos casos foram atribuídos a causas secundárias, com destaque para hipercalciúria, má absorção de cálcio, hiperparatireoidismo, deficiência de vitamina D, hipertireoidismo, doença de Cushing e hipercalcemia, de acordo com Tannenbaum et al. 41 Médicos e pacientes devem conversar sobre o melhor tratamento a ser adotado com base nas necessidades e preferências dos pacientes. As vantagens e desvantagens das alternativas de tratamento devem ser abordadas. Se os pacientes não conseguirem compreender as informações, seus cuidadores devem participar da conversa, segundo as diretrizes do National Institute for Health and Care Excellence (NICE), do Reino Unido. 42

Os medicamentos para osteoporose têm diferentes mecanismos de ação para fortalecer os ossos e reduzir o risco de fraturas. Bifosfonatos inibem osteoclastos, o que preserva a DMO. Moduladores seletivos do receptor de estrogênio ( *selective estrogen receptor modulators* , SERMs, em inglês) como o raloxifeno atuam como agonistas em alguns tecidos ósseos. A terapia de reposição hormonal (TRH) utiliza estrogênio e progesterona em mulheres na pós-menopausa. Inibidores do ligante do receptor ativador do fator nuclear kappa-B ( *receptor activator of nuclear factor Kappa-B ligand* , RANKL, em inglês), como denosumabe, reduzem a reabsorção óssea. Teriparatida é um análogo do PTH que estimula a formação óssea. Medicamentos como romosozumabe estimulam a formação óssea ao inibir a esclerostina, uma proteína produzida por osteócitos. Além disso, cálcio e vitamina D são frequentemente recomendados para manter a saúde óssea. A escolha do tratamento depende de fatores individuais, e deve ser orientada por médico especializado.

O tratamento de primeira linha para mulheres na pós-menopausa reside nos bisfosfonatos, seja alendronato ou risedronato. Bifosfonatos intravenosos ou denosumabe são recomendados para pacientes que não toleram bifosfonatos orais. A TRH com raloxifeno ou teriparatida também pode ser levada em consideração. 42

**Fig. 5:**
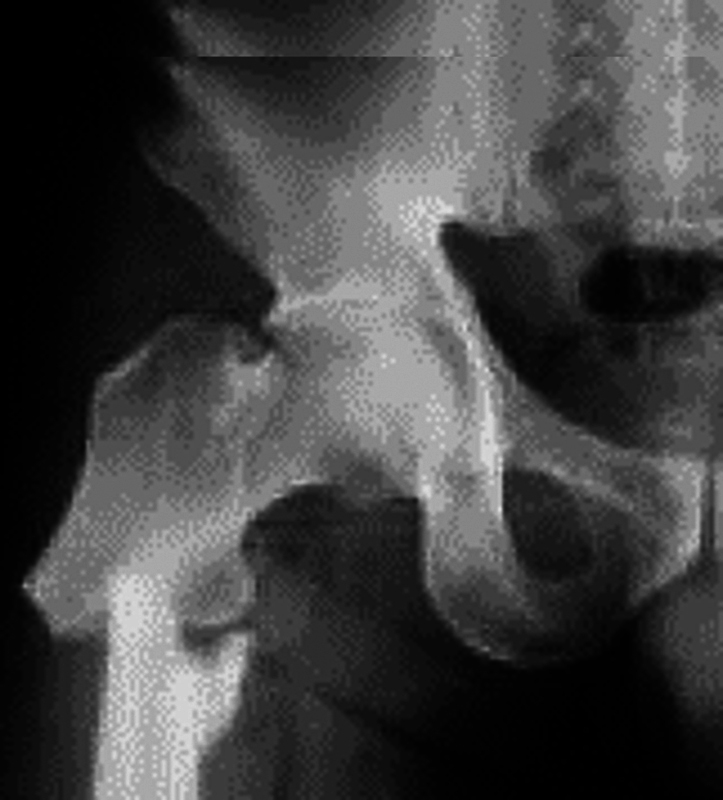
Radiografia que mostra fratura subtrocantérica direita atípica de fêmur em idoso, após trauma mínimo.

Fraturas femorais atípicas podem ocorrer após trauma mínimo, especialmente em casos de uso crônico de bifosfonatos ( Fig. 5 ), e a recomendação é interromper o uso desses medicamentos e considerar o tratamento com teriparatida. A suplementação de vitamina D e cálcio deve ser avaliada. O uso prolongado de bifosfonatos prejudica a qualidade óssea, pois inibe a remodelação óssea celular. A bilateralidade do problema deve ser considerada ao se avaliar o lado oposto. 43 Apesar dos cuidados oferecidos atualmente, o prognóstico para essas fraturas ainda é desfavorável, com um tempo de consolidação que varia de 12 a 60 meses. 43 Alendronato e risedronato são tratamentos de primeira linha para homens, ao passo que ácido zoledrônico, denosumabe ou teraparatida são alternativas. Os escores T da DMO do colo femoral em homens devem ser baseados no banco de dados de referência feminino do National Health and Nutrition Examination Survey (NHANES), dos Estados Unidos. 44

Recomenda-se a terapia de proteção óssea para indivíduos com mais de 70 anos que fazem uso de doses elevadas de glicocorticoides, especialmente prednisolona acima de 7,5 mg/dia. Mulheres na pós-menopausa e homens mais jovens em uso de doses elevadas também devem considerar essa terapia. Para aqueles com alto risco de fraturas, a terapia de proteção óssea deve ser iniciada no início do tratamento com glicocorticoides, e alendronato e risedronato são os tratamentos de primeira linha. 44

### Selecionando Diretrizes e Recomendações

A revisão sistemática de Solomon et al. 45 destaca a consistência nas recomendações das diretrizes para osteoporose, especificamente as da American Association of Clinical Endocrinologists (AACE) e do American College of Endocrinology (ACE). 24 Para o manejo da osteoporose na pós-menopausa, as opções de tratamento de primeira linha incluem alendronato, risedronato, ácido zoledrônico ou denosumabe para pacientes sem fraturas prévias por fragilidade ou com risco moderado de fratura. Para os pacientes com fraturas prévias por fragilidade ou alto risco de fratura, recomenda-se denosumabe, teriparatida ou ácido zoledrônico como tratamento de primeira linha. Alternativamente, alendronato e risedronato são opções de tratamento. O American College of Physicians (ACP) 37 sugere o uso de alendronato, risedronato, ácido zoledrônico ou denosumabe por 5 anos para mulheres com DMO baixa e osteoporose. 37 Recomenda-se a individualização do tratamento em mulheres de 65 anos ou mais, considerando fatores como risco *versus* benefício, preferências do paciente, perfil de risco de fratura e custos. O tratamento com terapia de estrogênio na menopausa, terapia combinada de estrogênio e progestágeno ou raloxifeno não é amplamente recomendado, e o monitoramento da DMO ao longo de 5 anos é controverso. 41 44 45

A escolha entre medicamentos para osteoporose muitas vezes se baseia em preferências pessoais, conveniência e adesão ao esquema posológico. A duração ideal do tratamento com bifosfonatos, devido à incerteza relacionada à fratura femoral atípica, é debatida, mas a maioria dos especialistas sugere uma pausa após 5 anos, especialmente para pacientes sem fraturas por fragilidade e com manutenção da densidade óssea. 46 Embora os medicamentos reduzam a probabilidade de fraturas, eles não eliminam todos os riscos, e tratamentos complementares como exercícios físicos e fisioterapia são igualmente importantes no manejo da osteoporose. 46

### Manejo Não Farmacológico e Exercício

O manejo não farmacológico da osteoporose abrange a ingestão adequada de cálcio e vitamina D, exercícios com levantamento de peso, cessação do tabagismo, limitação do consumo de álcool/cafeína e técnicas de prevenção de quedas. A vitamina D desempenha um papel crucial na absorção de cálcio e na saúde óssea, e são recomendadas doses diárias mais baixas para reduzir o risco de quedas. 46 A fisioterapia é essencial para fortalecer músculos, melhorar movimentos articulares e reduzir sarcopenia, e promove uma marcha mais estável. Implementar rotinas de equilíbrio nos exercícios pode contribuir significativamente para prevenir quedas, um fator comum de fraturas em pacientes com osteoporose.

### Prevenção de Quedas

Quedas na população idosa resultam de uma interação complexa entre fatores intrínsecos e extrínsecos, sendo que os riscos ambientais contribuem para cerca de 40% das quedas. Fatores como privação socioeconômica aumentam a incidência de fraturas, e mais de 90% delas resultam de quedas de baixa energia, associadas a taxas significativas de mortalidade. 47 48 49 50 O envelhecimento, as comorbidades e a alta hospitalar prematura estão ligados ao aumento das taxas de mortalidade. Estudos 2 3 4 17 48 indicam que metade das quedas ocorre durante a caminhada, muitas vezes devido a tropeços ou escorregões, ao passo que fatores ambientais, como superfícies irregulares, pisos molhados e objetos soltos, são desencadeadores frequentes. A tendência crescente a quedas ao ar livre também está relacionada a fatores extrínsecos.

Mulheres que caminham rapidamente e tropeçam têm maior probabilidade de cair para a frente, o que pode resultar em fraturas no punho, no braço e no cotovelo, ao passo que quedas laterais podem causar fraturas no quadril e na coluna vertebral. 16 17 18 19 Prevenir fraturas envolve a manutenção de ruas bem pavimentadas, a colocação de avisos em áreas com população idosa e o investimento em políticas públicas de saúde. Sapatos com saltos altos e largos desempenham um papel significativo em tais quedas, e recomenda-se a limitação de seu uso. 37 49 50 51 52 53 Medicamentos e problemas de visão e de audição podem afetar o equilíbrio, e programas de equilíbrio são benéficos para a melhora da estabilidade. O *tai chi chuan* se destaca como um excelente exercício para aumentar a estabilidade e prevenir quedas em idosos. 5 37 42 43

No teste Timed Up and Go, 54 o paciente levanta-se de uma cadeira, caminha 5 metros, retorna, e sentando-se novamente sem usar os braços como apoio. Como medida profilática, recomenda-se a prescrição de muletas ou andadores para pacientes incapazes de realizar o teste sem apoio ou que apresentam risco de queda. Além disso, a violência doméstica pode ser uma causa de quedas em idosos e, quando confirmada, requer notificação compulsória.

## Considerações Finais

A osteoporose aumenta a suscetibilidade dos indivíduos a fraturas e leva a taxas substanciais de morbidade e mortalidade em idosos. O diagnóstico inclui teste de densitometria óssea, que avalia a DMO em ossos com maior probabilidade de fratura, como a parte inferior da coluna (vértebras lombares), o colo do fêmur e os ossos do antebraço. A osteoporose é classificada em primária e secundária (causada por diversas comorbidades e/ou medicamentos).

A osteoporose é frequentemente diagnosticada após a primeira fratura clínica dos pacientes; a maioria dos pacientes com fraturas osteoporóticas necessita de tratamento cirúrgico ortopédico. A osteoporose está geralmente relacionada com a idade; é subdiagnosticada porque permanece assintomática (doença silenciosa) por vários anos até o desenvolvimento de fraturas. A FRAX calcula uma probabilidade de ocorrência de uma fratura osteoporótica grave num período de 10 anos. É necessário desenvolver políticas de saúde pública focadas na prevenção da fragilidade secundária, bem como investir constantemente em políticas de saúde pública para proporcionar benefícios sociais e económicos à população idosa.

O tratamento da osteoporose é multidisciplinar e deve incluir avaliação periódica do paciente, prescrição de novos medicamentos ósseos, e orientação sobre como evitar quedas. Os bifosfonatos são o medicamento mais utilizado, mas seu uso prolongado pode causar fratura femoral atípica, e os pacientes devem ser informados desse risco potencial.

**Tabela 2:** Escores T e critérios de diagnóstico da Organização Mundial de Saúde para osteoporose 2

Escore T*	
> −1	Dendidade óssea normal
Entre −1 e −2,5	Essa pontuação é sinal de osteopenia; esta condição apresenta densidade óssea abaixo do normal e pode levar à osteoporose.
< −2,5	Essa densidade óssea indica que a pessoa provavelmente terá osteoporose.

**Nota:** *Os valores de referência variam dependendo da localização geográfica.
